# Prevalence and Determinants of Metabolic Syndrome Among Adults (18–60 Years) in Urban and Rural South India: A Community-Based Cross-Sectional Study

**DOI:** 10.7759/cureus.84384

**Published:** 2025-05-19

**Authors:** Revathy Prasad Bidhu, Aswani Muraleedharan, Roy A Daniel, B.N. Surya

**Affiliations:** 1 Major Trauma, St Mary’s Hospital, Imperial College Healthcare National Health Service (NHS) Trust, London, GBR; 2 Trauma and Orthopaedics, Pilgrim Hospital, United Lincolnshire Hospitals National Health Service (NHS) Trust, Boston, GBR; 3 Community Medicine, Employee State Insurance Corporation (ESIC) Medical College and Hospital, Chennai, IND; 4 Community Medicine, Chettinad Hospital and Research Institute, Chettinad Academy of Research and Education, Chennai, IND

**Keywords:** community-based study, india, metabolic syndrome, obesity, prevalence, risk factors, rural, urban

## Abstract

Background

Metabolic syndrome (MetS) constitutes a multifaceted clinical condition characterised by a constellation of metabolic risk factors including central obesity, atherogenic dyslipidemia, elevated blood pressure, and impaired glucose metabolism, which synergistically elevate the risk of cardiovascular disease (CVD) and type 2 diabetes mellitus (T2DM). Rapid shifts in lifestyle have notably elevated the incidence of MetS in India, particularly in urban regions. Yet, sparse comparative data is available regarding the urban-rural differences in MetS prevalence within Tamil Nadu. Thus, this research sought to assess the prevalence of MetS and to identify associated risk factors among adults residing in urban and rural locales of Tamil Nadu.

Methods

Employing a multistage random sampling approach, this community-centric cross-sectional study encompassed 1,000 adults, evenly divided between urban (n=500) and rural (n=500) settings. Data were gathered through structured face-to-face interviews, anthropometric assessments, and biochemical analyses. The National Cholesterol Education Program Adult Treatment Panel III (NCEP ATP III) guidelines were utilised to define MetS. Analytical methods included descriptive statistics, chi-square tests, independent samples t-tests, and multivariate logistic regression modelling to pinpoint the critical determinants of MetS.

Results

The cumulative prevalence of MetS was 31.6% (95% confidence interval (CI): 28.7%-34.6%; 316 participants), with a higher prevalence in urban areas at 34.8% (95% CI: 31.9%-37.8%; 174 participants) compared to 28.4% in rural areas (95% CI: 25.6%-31.3%; 142 participants). The study identified significant predictors of MetS, such as age 40 years or above (adjusted odds ratio (AOR): 2.2, p<0.01), female sex (AOR: 2.8, p<0.01), tobacco smoking (AOR: 2.7, p<0.01), sedentary lifestyle (AOR: 2.7, p=0.02), minimal fruit intake (AOR: 4.4, p=0.01), habitual consumption of fried foods (AOR: 5.3, p=0.01), existing diabetes mellitus (AOR: 3.2, p=0.02), and obesity (AOR: 2.3, p=0.03).

Conclusion

Urban populations exhibited a higher MetS prevalence, likely attributed to modifiable dietary patterns and lifestyle choices. Regular screening initiatives, lifestyle adjustments, and targeted nutritional interventions emerge as vital preventive and therapeutic measures. Public health strategies should emphasise early detection and encourage behavioural modifications, specifically targeting at-risk populations.

## Introduction

Metabolic syndrome (MetS) is typically diagnosed when an individual presents with at least three out of five defined clinical abnormalities, which include increased waist circumference (indicative of central obesity), elevated serum triglyceride levels, reduced high-density lipoprotein cholesterol, elevated arterial blood pressure, and raised fasting plasma glucose concentrations. This criterion-based diagnostic approach most commonly outlined by the National Cholesterol Education Program Adult Treatment Panel III (NCEP ATP III) and the International Diabetes Federation (IDF) provides a standardised framework for identifying individuals at increased cardiometabolic risk. Nonetheless, there remains ongoing scientific debate as to whether metabolic syndrome constitutes a distinct clinical entity or merely reflects a statistical aggregation of interrelated risk factors for cardiovascular disease and type 2 diabetes mellitus [1,2]. MetS, defined by international criteria such as NCEP ATP III and IDF, is recognised as a major contributor to global morbidity and mortality [3]. The prevalence of MetS has been increasing worldwide, particularly in low- and middle-income countries like India, where urbanisation, dietary transitions, and sedentary lifestyles have accelerated its burden. Recent epidemiological studies estimate that approximately 30% of Indian adults meet the criteria for MetS, with significant variation across regions and population subgroups [4]. Recent evidence indicates that Tamil Nadu, especially urban centres like Chennai, shows a markedly high burden of MetS. A multisite population-based study reported a prevalence of 32% (95% confidence interval (CI): 30%-34%) among adults in Chennai using the NCEP ATP III criteria. Further supporting this, a systematic review and meta-analysis found a pooled MetS prevalence of 30% (95% CI: 28%-33%) across India, with urban areas having a significantly higher prevalence (32%; 95% CI: 29%-36%) compared to rural settings (22%; 95% CI: 20%-25%). Notably, even rural regions traditionally perceived as lower risk are increasingly witnessing convergence with urban prevalence patterns, likely due to shifts in diet, physical inactivity, and lifestyle transitions associated with rural-to-urban migration and modernisation [5-7]. Although the burden of MetS is increasing, there is limited comparative data on the disparities in prevalence between urban and rural areas within the state, highlighting the need for further research.

The urban-rural dichotomy in MetS prevalence in India is well established, with urban populations consistently exhibiting higher prevalence than rural counterparts [8]. This difference arises mainly from higher intake of processed and convenience foods, sedentary behaviour, and elevated psychosocial stress typically observed in urban areas; conversely, rural communities have traditionally adhered to more physically demanding lifestyles coupled with conventional dietary practices [8]. However, the epidemiological transition in rural India, characterised by mechanisation of labour, increased access to energy-dense foods, and shifting occupational patterns, has led to a convergence in metabolic risk factors [9]. Studies in Tamil Nadu have corroborated this shift, with rural communities, particularly those in proximity to urban centres, demonstrating rising rates of metabolic syndrome comparable to urban populations. Notably, these studies employed standardised diagnostic criteria such as NCEP ATP III or IDF, enhancing the comparability of prevalence estimates across settings [5,6]. Given these trends, it is imperative to assess the current burden of MetS in both urban and rural settings to identify population-specific risk factors and inform targeted public health interventions.

Despite the significant public health implications of MetS, there is a paucity of epidemiological studies that systematically compare its prevalence between urban and rural populations in Tamil Nadu. The absence of such data limits the ability to design context-specific prevention and intervention strategies, as most existing recommendations are based on either national-level estimates or isolated urban or rural studies. The aim of this research is to close this knowledge gap by delivering current estimates of MetS prevalence across urban and rural communities in Tamil Nadu, employing uniform diagnostic standards and rigorous epidemiological approaches. The findings will offer critical insights into the distribution of MetS and associated risk factors, contributing to evidence-based policymaking and developing targeted metabolic health interventions for diverse population groups.

## Materials and methods

A community-based cross-sectional study was carried out among adults aged 18-60 years in selected urban and rural areas of Tamil Nadu. Urban participants were recruited from Chennai, a metropolitan city with a diverse socioeconomic population, while rural participants were selected from villages in the Kanchipuram district in Tamil Nadu. The study areas were chosen to reflect differences in lifestyle, dietary habits, and healthcare access between urban and rural settings. The study was conducted between February 2024 and April 2024, which included participant recruitment, data collection, and biochemical investigations. Inclusion criteria were as follows: Adults aged 18-60 years who were permanent residents of the selected urban and rural areas for at least six months, and individuals who provided informed consent to participate in the study. Pregnant and lactating women, individuals with diagnosed endocrine disorders (except diabetes mellitus), and patients on long-term corticosteroid therapy were excluded from the study. Sample size was calculated with the standard formula $n = \frac{Z^2 \cdot p \cdot (1 - p)}{d^2}$. Since multistage sampling was employed and a design effect (DEFF) of 1.03 was applied to adjust for intra-cluster correlation, the sample size increased to approximately 999. After rounding, the final sample size was fixed at 1,000 participants, evenly distributed between urban and rural areas (n=500 each).

In the urban setting, a multistage random sampling strategy was implemented to ensure adequate representation. Two administrative zones were randomly selected from the Chennai Metropolitan Area, followed by the random selection of two wards from each zone. Within each selected ward, a central, well-recognised public landmark (e.g., a bus stop, school, or temple) was predetermined as the reference point for field navigation. The initial direction for household enumeration was selected using a random directional technique, wherein a bottle was rotated on flat ground at the landmark. The direction indicated upon cessation of rotation was followed as the initial path. In case of obstruction (e.g., locked access road or physical barrier), the adjacent clockwise path was chosen as a backup. The first household along the selected path was enrolled as the starting point. Thereafter, every 20th household was approached until the desired sample quota was achieved for that ward. In households with more than one eligible participant (aged 18-60 years), the Kish Grid Method was applied to randomly select a single individual, ensuring equal probability of inclusion.

Similarly, in the rural setting, two villages were randomly chosen from the Kanchipuram district. The total number of households in each village was determined using records obtained from the block's primary health centre (PHC). In cases where a selected household was unavailable, locked after two consecutive visits at different times of the day, or declined to participate, the immediate next household on the right was approached as a replacement. This substitution was made strictly according to the pre-defined protocol to maintain the sampling interval and avoid arbitrary replacements. Replacement households were recorded and reported separately for quality assurance. This approach aligns with standard non-response handling methods in large-scale household surveys to minimise selection bias and preserve representativeness. Additionally, field investigators maintained detailed logs of visited households to track response rates and avoid duplication or clustering effects in participant selection.

Information/data was gathered via in-person interviews conducted with the aid of a pre-validated semi-structured questionnaire, alongside clinical assessments and laboratory tests. This survey tool documented various sociodemographic factors such as age, sex, educational background, occupation, income level, and socioeconomic classification was conducted using the updated BG Prasad Scale (2023), which categorises individuals into Class I to Class V based on monthly per capita income thresholds, with Class I representing the highest socioeconomic status (SES) (≥INR 8,822) and Class V the lowest (<INR 1,323) [10]), lifestyle factors (physical activity assessed using the Global Physical Activity Questionnaire (GPAQ) [11], dietary habits, smoking, and alcohol consumption), and family history of diabetes, hypertension, and cardiovascular diseases (CVD). Anthropometric measurements were recorded, including waist circumference and body mass index (BMI). Blood pressure was measured in a sitting position using a calibrated aneroid sphygmomanometer, taking the average of two readings. Biochemical assessments included measuring fasting blood sugar levels (utilising the glucose oxidase technique) along with a comprehensive lipid profile, which encompassed total cholesterol, high-density lipoprotein (HDL), low-density lipoprotein (LDL), and triglyceride levels.

To ensure data quality, all anthropometric measurements were standardised using calibrated instruments, the biochemistry machine in the lab was checked daily using Levey-Jennings (LJ) plot, digital weighing scales and stadiometers were routinely checked for accuracy, and a separate log was maintained. The structured questionnaire underwent pre-testing on 10% of the sample outside the study area to assess clarity, consistency, and feasibility, incorporating modifications based on feedback. Data collectors were postgraduate students who received standardised training for one week from the study investigators to minimise measurement bias. Internal quality control procedures included daily calibration of digital sphygmomanometers against a mercury standard and verification of anthropometric instruments (weighing scales and stadiometers) at the start and midpoint of each data collection day. Laboratory-based biochemical analyses were validated using external quality assurance schemes and blinded duplicate samples, analysed in 10% of cases to assess intra-laboratory reproducibility. Supervisory teams performed weekly field audits, reviewed randomly selected forms for accuracy, and ensured protocol adherence through on-site re-measurements in 5% of the participants. All deviations were logged and addressed in real time to maintain methodological consistency. The primary outcome measure was the prevalence of MetS, defined according to NCEP ATP III criteria. As per this definition, a diagnosis of MetS is made when an individual presents with three or more of the following five components: (1) abdominal obesity - waist circumference ≥102 cm in men or ≥88 cm in women; (2) hypertriglyceridemia - triglycerides ≥150 mg/dL, (3) low HDL cholesterol - <40 mg/dL in men or <50 mg/dL in women; (4) elevated blood pressure - systolic ≥130 mmHg and/or diastolic ≥85 mmHg, and (5) impaired fasting glucose - fasting plasma glucose ≥100 mg/dL [3]. Ethical approval was obtained from the Institutional Ethics Committee (IEC; No: IHEC-II/0842/24) before study initiation. Written informed consent was obtained from all participants prior to enrolment. Participant data privacy and anonymity were safeguarded throughout the study. Individuals identified with MetS or any of its components received appropriate counselling and were directed to the nearest primary healthcare facilities for continued evaluation and treatment.

Statistical analysis

Data were collected using EpiCollect5, a free and open-source mobile and web application developed by the Centre for Genomic Pathogen Surveillance (CGPS), based at the University of Oxford and the Wellcome Sanger Institute. The platform is accessible at https://five.epicollect.net. The data were analysed using Stata version 14 (Stata Statistical Software Release 14, StataCorp LLC, College Station, TX, USA). Descriptive statistics were used to summarise participant characteristics, and prevalence estimates were reported as percentages with 95% confidence intervals. A comparative assessment between urban and rural groups was carried out employing chi-square tests for categorical data and independent t-tests for continuous measurements. To determine independent predictors of MetS, multivariate logistic regression was utilised for variables that demonstrated a p-value below 0.05 in the bivariate analysis. The results were expressed as adjusted odds ratios (AOR) along with 95% confidence intervals. Statistical significance was set at a p-value threshold of less than 0.05.

## Results

We obtained data from 1,000 participants, 500 each from urban and rural areas and included all the participants in the final analysis. Gender distribution was nearly equal, with males comprising 51.2% and females 48.8%, with no significant difference between urban and rural populations (p=0.949). Similarly, the age distribution was comparable, with 52.6% of participants aged ≥40 years and 47.4% aged <40 years, showing no significant difference (p=0.375). However, significant differences were noted in education and socioeconomic status (p<0.01). Urban participants had higher graduate rates (35.6% vs. 23.8%), while rural participants had more illiteracy (17% vs. 12%). Socioeconomic disparities were evident, with urban areas having more upper-class individuals (25.2% vs 12.6%), whereas middle-class representation was higher in rural areas (57.6% vs 47.8%), as shown in Table 1.

**Table 1:** Sociodemographic Characteristics of Study Participants (Urban vs. Rural) Chi-square (χ²) test was employed. A p-value < 0.05 is considered statistically significant.

Variable	Urban (n=500)	Rural (n=500)	Total (n=1000)	P-Value (Test Statistic)
Gender				=0.949 (χ²=0.004)
Male (%)	256 (51.2%)	256 (51.2%)	512 (51.2%)	
Female (%)	244 (48.8%)	244 (48.8%)	488 (48.8%)	
Age Group				=0.375 (χ²=0.789)
<40 years (%)	230 (46.0%)	244 (48.8%)	474 (47.4%)	
≥40 years (%)	270 (54.0%)	256 (51.2%)	526 (52.6%)	
Education Level				<0.01 (χ²=9.622)
Up to Primary Schooling Level (%)	60 (12.0%)	85 (17.0%)	145 (14.5%)	
Graduate (%)	178 (35.6%)	119 (23.8%)	297 (29.7%)	
Socioeconomic Status				<0.01 (χ²=12.451)
Upper Class (%)	126 (25.2%)	63 (12.6%)	189 (18.9%)	
Middle Class (%)	239 (47.8%)	288 (57.6%)	527 (52.7%)	

**Figure 1:**
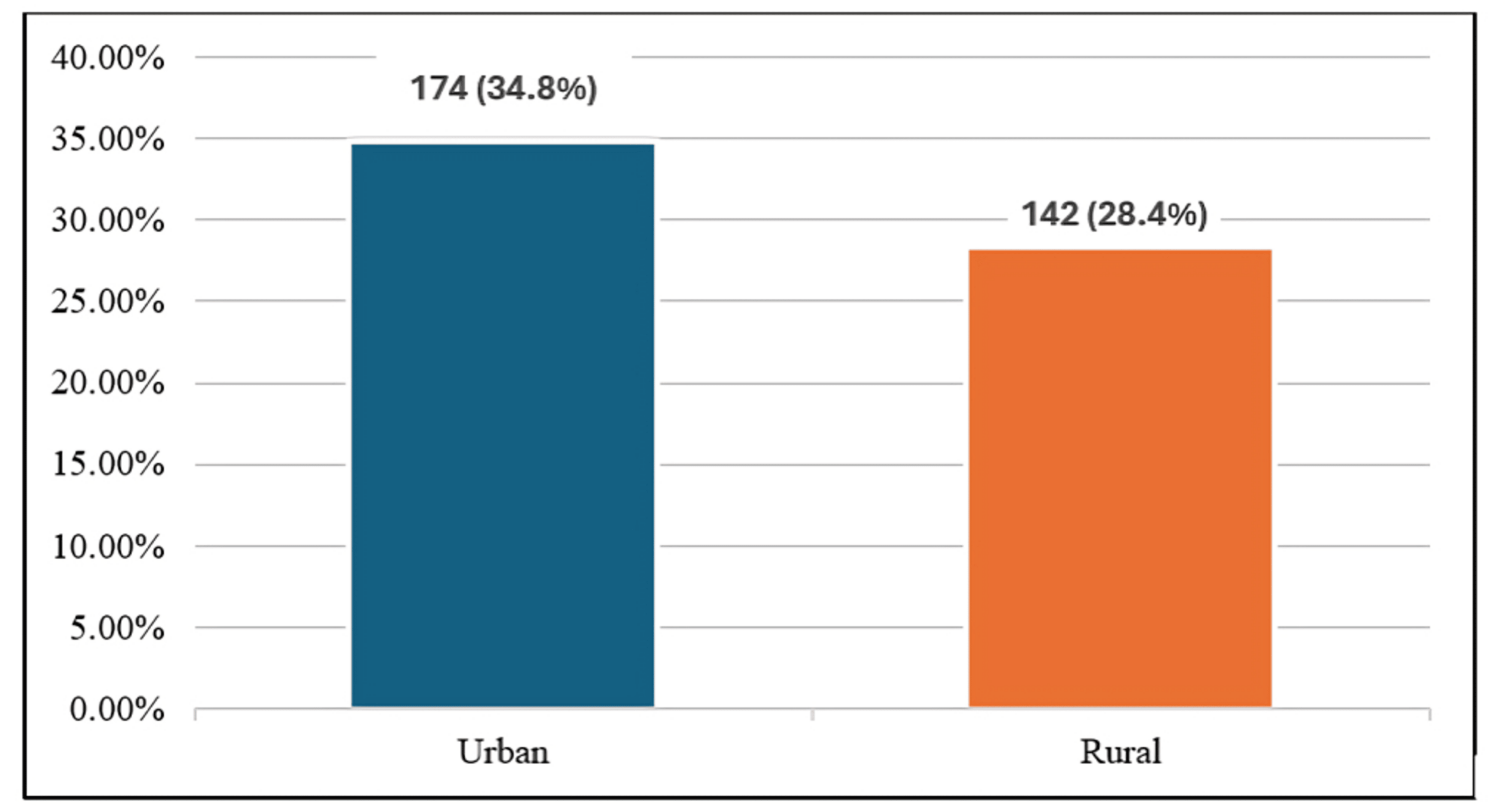
Prevalence of Metabolic syndrome among the study participants (N=1,000) The figure illustrates the prevalence of metabolic syndrome (MetS) among urban and rural participants. MetS was identified based on the NCEP ATP III criteria. The prevalence in urban participants (34.8%, n=174) and rural participants (28.4%, n=142). NCEP ATP III: National Cholesterol Education Program Adult Treatment Panel III.

**Figure 2:**
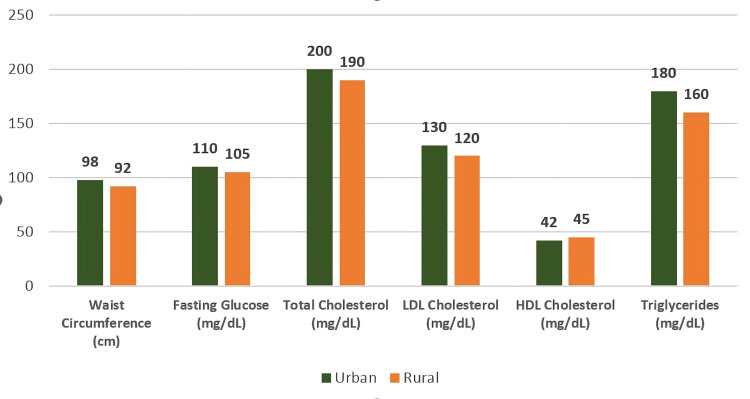
Distribution of Metabolic Parameters (Urban vs. Rural) Statistical test used: Independent samples t-test. Reference ranges: waist circumference: <90 cm; fasting glucose: <100 mg/dL; total cholesterol: <200 mg/dL; LDL cholesterol: <130 mg/dL; HDL cholesterol: ≥40 mg/dL; triglycerides: <150 mg/dL. P-values: waist circumference (cm): p=0.007; fasting glucose (mg/dL): p=0.02; total cholesterol (mg/dL): p<0.001; LDL cholesterol (mg/dL): p<0.001; HDL cholesterol (mg/dL): p=0.15; triglycerides (mg/dL): p=0.05. A p-value < 0.05 is considered statistically significant.

The overall prevalence of MetS among the participants was recorded at 31.6% (95% CI: 28.7%-34.6%). When comparing locations, urban regions exhibited a higher MetS prevalence of 34.8% (95% CI: 31.9%-37.8%), whereas rural areas showed a lower rate of 28.4% (95% CI: 25.6%-31.3%). This data is illustrated in Figure 1, with Figure 2 presenting the distribution of specific parameters across urban and rural groups.

Individuals diagnosed with MetS exhibited markedly higher levels of waist circumference, fasting blood glucose, total cholesterol, LDL cholesterol, and triglycerides. Conversely, their HDL cholesterol levels were notably lower than those of participants without MetS. These differences across all measured parameters were statistically significant (p<0.001), as detailed in Table 2.

**Table 2:** Comparison of Metabolic Parameters Between Participants With and Without MetS t-test applied. A p-value < 0.05 is considered statistically significant.

Parameter	With MetS (n=316)	Without MetS (n=684)	P-Value	Test Statistic (t-value)
Waist Circumference (cm)	102.5±8.4	88.3±7.2	<0.001	23.50
Fasting Blood Glucose (mg/dL)	112.4±18.6	91.2±10.4	<0.001	16.78
Total Cholesterol (mg/dL)	215.6±28.3	183.7±24.1	<0.001	15.04
LDL Cholesterol (mg/dL)	138.5±19.8	113.2±17.6	<0.001	17.90
HDL Cholesterol (mg/dL)	38.9±5.4	51.7±6.2	<0.001	-26.05
Triglycerides (mg/dL)	178.4±36.5	129.6±27.4	<0.001	20.12

Bivariate and multivariate logistic regression analysis identified age ≥40 years (AOR: 2.2, p<0.01) and female gender (AOR: 2.8, p<0.01) as significant risk factors for MetS. Among lifestyle factors, smoking (AOR: 2.7, p<0.01), physical inactivity (AOR: 2.7, p=0.02), frequent fried food consumption (AOR: 5.3, p=0.01), and low fruit consumption (AOR: 4.4, p=0.01) were significantly associated with MetS. Metabolic parameters such as diabetes (AOR: 3.2, p=0.02), obesity (AOR: 2.3, p=0.03), hypercholesterolemia ≥200 mg/dl (AOR: 2.4, p=0.01), and hypertriglyceridemia ≥150 mg/dl (AOR: 2.3, p<0.01) were independently associated with MetS as shown in Table 3. The multivariate logistic regression model demonstrated acceptable fit, as indicated by the Hosmer-Lemeshow goodness-of-fit test (χ² = 7.62, df = 8, p = 0.472). The model’s explanatory power, assessed using the Nagelkerke pseudo R², was 0.022, indicating modest variance explained. These findings support the statistical adequacy of the model in estimating independent associations with MetS.

**Table 3:** Multivariate Logistic Regression Analysis for Determinants of Metabolic Syndrome (MetS) A p-value < 0.05 is considered statistically significant.

Variable	Adjusted Odds Ratio (AOR)	95% Confidence Interval	p-value
Age≥40 years	2.2	1.6-3.1	<0.01
Female Gender	2.8	1.9-4.0	<0.01
Tobacco Smoking	2.7	1.7-4.2	<0.01
Physical Inactivity	2.7	1.3-5.6	0.02
Frequent Fried Food Consumption	5.3	2.1-13.1	0.01
Low Fruit Consumption	4.4	1.8-10.8	0.01
Diabetes Mellitus	3.2	1.2-8.5	0.02
Obesity (BMI ≥30 kg/m²)	2.3	1.1-4.8	0.03
Hypercholesterolemia ≥200 mg/dL	2.4	1.3-4.4	0.01
Hypertriglyceridemia ≥150 mg/dL	2.3	1.5-3.6	<0.01

## Discussion

This community-based cross-sectional study assessed MetS prevalence and determinants among 1,000 adults (500 urban, 500 rural) in Tamil Nadu, India. The study found an overall MetS prevalence of 31.6%, with a significantly higher prevalence in urban areas (34.8%) than in rural areas (28.4%).

The current study found that urban residents had a higher prevalence of MetS than their rural counterparts, a pattern consistently observed in various studies across India. This urban-rural difference can be attributed to lifestyle factors, dietary patterns, and socioeconomic status, all of which influence metabolic health. A multisite study in India demonstrated that the urban populations had a significantly higher burden of MetS, primarily driven by increased obesity, sedentary lifestyles, and greater consumption of processed and high-calorie foods [12]. The process of urbanisation has led to a dietary transition from fibre-dense traditional meals towards consumption patterns dominated by high-fat, sugar-rich, and processed foods. This nutritional shift, combined with declining physical activity levels driven by mechanised work environments and occupational transformations, has collectively fuelled the elevated prevalence of MetS observed in urban populations.

A study reported a MetS prevalence of 35.8% (NCEP ATP III criteria), with a significantly higher proportion of cases in urban areas than rural regions [13]. Notably, this study adjusted for key confounding variables such as age, sex, and physical activity. Urban predominance of MetS can be linked to higher socioeconomic status (SES), which often correlates with greater access to energy-dense foods, increased screen time, and reduced physical exertion [13,14]. High SES groups are more likely to engage in sedentary jobs and have access to convenience-based diets, both of which increase the risk of developing MetS. Conversely, rural populations have traditionally exhibited lower MetS prevalence, as seen in a study from Western Uttar Pradesh, which found an overall MetS prevalence of only 11.7% in rural communities. Rural residents typically have higher levels of occupational and non-occupational physical activity, particularly in agricultural labour and daily commuting, which protects against obesity and metabolic dysfunction [15].

Our study findings reinforce this established urban-rural MetS divide. MetS is becoming increasingly prevalent in rural populations due to ongoing urbanisation and lifestyle transitions, but rural communities still exhibit a relatively lower burden than urban areas [16]. It is likely due to significant physical activity, continued adherence to traditional diets, and lower levels of processed food consumption. However, as rural areas experience improved economic development, urban influences, and dietary shifts, the MetS burden is expected to rise further, necessitating early public health interventions to mitigate this trend. We also identified socioeconomic status as a crucial determinant of MetS. Individuals from higher socioeconomic strata were more likely to have MetS, as reported in previous research [17]. Urbanisation and affluence often lead to increased consumption of processed and high-fat foods, a shift toward sedentary occupations, and higher rates of obesity. In contrast, lower socioeconomic groups in rural settings may be less prevalent due to physically demanding labour and traditional, less calorie-dense diets.

The current findings indicate a higher prevalence of MetS among women than men, particularly in urban settings. These findings align with a study which demonstrated a significant MetS burden in urban women due to hormonal factors, lifestyle changes, and central obesity. Biological plausibility suggests that estrogen fluctuations, particularly post-menopause, contribute to increased visceral adiposity and dyslipidemia, predisposing women to MetS [18]. Age was another significant factor, with individuals aged 40 years and above showing a markedly higher prevalence, which is in line with previous findings indicating that MetS risk increases with age due to cumulative exposure to metabolic risk factors such as insulin resistance, hypertension, and dyslipidemia [13]. The progressive decline in metabolic efficiency and changes in adipose tissue distribution with ageing support this association.

Our study highlights central obesity as a primary driver of MetS, particularly in urban populations, which is consistent with existing literature suggesting that visceral fat accumulation leads to increased insulin resistance, chronic low-grade inflammation, and dyslipidemia. The pathophysiology of MetS is strongly linked to adipokine dysregulation, where excessive adipose tissue secretes pro-inflammatory cytokines such as tumour necrosis factor alpha (TNF-alpha) and interleukin-6 (IL-6), exacerbating insulin resistance and hypertension [19]. Lack of physical activity was a significant risk factor for MetS in our study, reinforcing findings from a cross-sectional study. Dietary patterns emerged as a crucial determinant, with higher consumption of processed and fried foods significantly associated with MetS. Studies have shown that high trans-fat and refined carbohydrate intake contributes to hyperglycaemia, dyslipidemia, and obesity. In contrast, traditional rural diets, which are fibre-rich and lower in refined sugars, may confer protective metabolic benefits [20]. These findings also emphasise the importance of integrating community-based dietary and lifestyle interventions with national initiatives such as the National Programme for Prevention and Control of Cancer, Diabetes, Cardiovascular Diseases and Stroke (NPCDCS), which targets early detection and lifestyle modification for MetS-related conditions. Such efforts should be harmonised with existing platforms like the Eat Right India campaign and the Fit India Movement to promote healthier food environments and physical activity at scale.

Study implications

The outcomes of this research carry important clinical relevance for the prompt identification, prevention, and control of MetS across both urban and rural settings. The elevated rates of MetS observed in urban populations, alongside critical risk contributors like obesity, sedentary lifestyles, poor dietary choices, and diabetes, underscore the necessity for focused lifestyle modification strategies tailored to these at-risk groups. Primary care physicians and public health practitioners should prioritise routine screening for MetS components, especially in individuals aged ≥40 years, females, and those with diabetes or hyperlipidaemia, as these groups were found to have the highest risk. Given the strong association between dietary patterns (fried food intake, low fruit consumption) and MetS, nutritional counselling and behaviour modification programs should be integrated into routine healthcare services. Additionally, community-based interventions, such as promoting physical activity, reducing smoking prevalence, and improving access to affordable healthy foods, are essential to mitigate the growing burden of MetS. These findings also emphasise the need for policy-level actions, including urban planning for active lifestyles, regulating processed foods, and expanding preventive healthcare services to curb India's rising metabolic disease epidemic.

Strengths and limitations

This study has several strengths, including its community-based design with a large, representative sample of urban and rural populations, allowing for a robust comparison of MetS prevalence and associated risk factors. The use of multistage random sampling minimised selection bias, and the application of standardised diagnostic criteria (NCEP ATP III) ensured comparability with national and international studies. Additionally, rigorous quality control protocols, including daily Levey-Jennings plots for laboratory assays and standardised anthropometric measurements, enhanced the reliability and reproducibility of data collection.

Nevertheless, certain limitations should be recognised. The cross-sectional design restricts causal inference and captures only a snapshot of exposure-outcome relationships. Furthermore, self-reported lifestyle factors (such as dietary intake, tobacco use, alcohol consumption, and physical activity) may be subject to recall and social desirability bias, potentially leading to underreporting of unhealthy behaviours, especially in culturally sensitive contexts. Although efforts were made to include demographically diverse urban and rural zones, the findings are drawn exclusively from Tamil Nadu and may not be fully generalisable to other Indian states, given the regional heterogeneity in cardiometabolic profiles, dietary practices, and healthcare infrastructure. Future studies should incorporate prospective cohort designs to elucidate temporal and causal pathways and intervention trials to assess the effectiveness of targeted lifestyle modifications. Linking such research to existing national initiatives, such as NPCDCS, could inform scalable strategies for reducing MetS burden across diverse Indian populations.

## Conclusions

This community-based study revealed a high prevalence of MetS among adults in Tamil Nadu, with urban populations disproportionately affected. Independent predictors included older age, female sex, tobacco use, physical inactivity, high intake of fried foods, low fruit consumption, obesity, diabetes, and dyslipidemia, highlighting the complex interplay of behavioural, demographic, and metabolic factors driving the syndrome. These findings reflect broader epidemiological transitions and support the implementation of risk-stratified screening, particularly for individuals aged ≥40 years and those with existing metabolic conditions.

To address the rising burden of MetS, integrated public health interventions are essential. Routine screening, lifestyle modification programs, and dietary counselling should be incorporated into primary healthcare delivery and aligned with national initiatives such as NPCDCS, Eat Right India, and the Fit India Movement. Future prospective studies and interventional trials are needed to establish causality and guide evidence-based prevention strategies tailored to India’s diverse regional populations.

## Appendices

1. BG Prasad Socioeconomic Classification (October 2023) questionnaire

The Modified BG Prasad Classification is a widely used socioeconomic scale for categorising families in urban and rural India based on per capita monthly income. This scale is periodically updated to reflect changes in the Consumer Price Index for Industrial Workers (CPI-IW), which tracks inflation and price changes across various commodities.

**Table 4:** Socioeconomic Classes and Income Ranges (October 2023)

Social Class	Description	Per Capita Monthly Income (Rs.)
Class I	Upper Class	INR 9,098 and above
Class II	Upper Middle Class	INR 4,549-9,097
Class III	Middle Class	INR 2,729-4,550
Class IV	Lower Middle Class	INR 1,365-2,728
Class V	Lower Class	Below INR 1,365

*Interpretation*: This classification helps determine the socioeconomic status (SES) of individuals and families, which is a significant determinant of health outcomes. Class I represents the highest socioeconomic status, while Class V represents the lowest.

2. Global Physical Activity Questionnaire (GPAQ) questionnaire

**Table 5:** Global Physical Activity Questionnaire (GPAQ)

Physical Activity					
Next I am going to ask you about the time you spend doing different types of physical activity in a typical week. Please answer these questions even if you do not consider yourself to be a physically active person. Think first about the time you spend doing work. Think of work as the things that you have to do such as paid or unpaid work, study/training, household chores, harvesting food/crops, fishing or hunting for food, seeking employment. (Insert other examples if needed.) In answering the following questions 'vigorous-intensity activities' are activities that require hard physical effort and cause large increases in breathing or heart rate, 'moderate-intensity activities' are activities that require moderate physical effort and cause small increases in breathing or heart rate.					
Questions		Response			Code
Activity at work					
1	Does your work involve vigorous-intensity activity that causes large increases in breathing or heart rate like [carrying or lifting heavy loads, digging or construction work] for at least 10 minutes continuously? [INSERT EXAMPLES] (USE SHOWCARD)	Yes	1		P1
		No	2	If No, go to P 4	
2	In a typical week, on how many days do you do vigorous-intensity activities as part of your work?	Number of days	└─┘		P2
3	How much time do you spend doing vigorous-intensity activities at work on a typical day?	Hours : minutes	└─┴─┘: └─┴─┘ hrs mins		P3 (a-b)
4	Does your work involve moderate-intensity activity that causes small increases in breathing or heart rate such as brisk walking [or carrying light loads] for at least 10 minutes continuously? [INSERT EXAMPLES] (USE SHOWCARD)	Yes	1		P4
		No	2	If No, go to P 7	
5	In a typical week, on how many days do you do moderate-intensity activities as part of your work?	Number of days	└─┘		P5
6	How much time do you spend doing moderate-intensity activities at work on a typical day?	Hours : minutes	└─┴─┘: └─┴─┘ hrs mins		P6 (a-b)
Travel to and from places					
The next questions exclude the physical activities at work that you have already mentioned. Now I would like to ask you about the usual way you travel to and from places. For example to work, for shopping, to market, to place of worship. [Insert other examples if needed.]					
7	Do you walk or use a bicycle (pedal cycle) for at least 10 minutes continuously to get to and from places?	Yes	1		P7
		No	2	If No, go to P 10	
8	In a typical week, on how many days do you walk or bicycle for at least 10 minutes continuously to get to and from places?	Number of days	└─┘		P8
9	How much time do you spend walking or bicycling for travel on a typical day?	Hours : minutes	└─┴─┘: └─┴─┘ hrs mins		P9 (a-b)
Recreational activities					
The next questions exclude the work and transport activities that you have already mentioned. Now I would like to ask you about sports, fitness and recreational activities (leisure) [insert relevant terms].					
10	Do you do any vigorous-intensity sports, fitness or recreational (leisure) activities that cause large increases in breathing or heart rate like [running or football,] for at least 10 minutes continuously? [INSERT EXAMPLES] (USE SHOWCARD)	Yes	1		P10
		No	2	If No, go to P 13	
11	In a typical week, on how many days do you do vigorous- intensity sports, fitness or recreational (leisure) activities?	Number of days	└─┘		P11
12	How much time do you spend doing vigorous-intensity sports, fitness or recreational activities on a typical day?	Hours : minutes	└─┴─┘: └─┴─┘ hrs mins		P12 (a-b)
Physical Activity (recreational activities) contd.					
Questions		Response			Code
13	Do you do any moderate-intensity sports, fitness or recreational (leisure) activities that causes a small increase in breathing or heart rate such as brisk walking,(cycling, swimming, volleyball)for at least 10 minutes continuously? [INSERT EXAMPLES] (USE SHOWCARD)	Yes 1 No 2 If No, go to P16			P13
14	In a typical week, on how many days do you do moderate-intensity sports, fitness or recreational (leisure) activities?	Number of days └─┘			P14
15	How much time do you spend doing moderate-intensity sports, fitness or recreational (leisure) activities on a typical day?	Hours : minutes	└─┘└─┘		P15 (a-b)
Sedentary behaviour					
The following question is about sitting or reclining at work, at home, getting to and from places, or with friends including time spent [sitting at a desk, sitting with friends, travelling in car, bus, train, reading, playing cards or watching television], but do not include time spent sleeping. [INSERT EXAMPLES] (USE SHOWCARD)					
16	How much time do you usually spend sitting or reclining on a typical day?	Hours : minutes	└─┘└─┘		P16 (a-b)
